# Acute Kidney Injury: A Blocked Diagnosis

**DOI:** 10.7759/cureus.88144

**Published:** 2025-07-17

**Authors:** Zhi Tian Chen, Lih Yin Chong, Irushna Perera, Rizuan Mohamed

**Affiliations:** 1 Care of Elderly Medicine, Mid and South Essex NHS Foundation, Basildon, GBR; 2 Acute Care Unit, Mid and South Essex NHS Foundation, Basildon, GBR

**Keywords:** acute care medicine, acute kidney injury, clinical nephrology, general nephrology, geriatrics and internal medicine, internal medicine

## Abstract

Acute kidney injury (AKI) is a common medical condition that we encounter in our daily clinical practice. However, in certain cases, ascertaining the underlying cause of the AKI could be challenging and requires a thorough history, focused investigations, and a high index of suspicion. We present the case of a 79-year-old gentleman who has been using a long-term urinary catheter and presented with AKI, which we found to be challenging to diagnose. In patients with a history of obstructive uropathy, particularly those using long-term catheters, a blocked catheter is the most probable cause of AKI. In this case, we found it challenging to establish a definitive diagnosis, given that the initial ultrasound scan of the kidneys failed to identify an obstruction. This necessitated broadening our differential diagnoses and resorting to second-line investigations such as a CT scan.

As the clinical suspicion remained high for an obstructive uropathy as the cause for the AKI, CT imaging was carried out, which revealed severe hydronephrosis with bilateral moderate hydroureter, a finding that was not picked up on routine sonography.

This case report highlights the importance of determining an accurate etiology of AKI to prevent delayed treatment and underscores the importance of clinical judgment in formulating a valid differential diagnosis.

## Introduction

Acute kidney injury (AKI) is a common medical condition that we encounter in our daily practice. It is defined as an abrupt decrease in kidney function, resulting in the retention of urea and other nitrogenous waste products and in the dysregulation of extracellular volume and electrolytes [1-3]. However, diagnosis and ascertaining the etiology of AKI can sometimes be challenging and not straightforward, especially during on-call periods when stress levels are high and resources are limited. We hereby present a challenging case of AKI in an elderly gentleman on a long-term catheter. 

The challenge we faced was determining the cause of the AKI in the face of a rapidly declining kidney function so that corrective treatment could be offered as early as possible. As is the norm, obstructive uropathy was high in our list of differential diagnoses for the AKI, given that he had a history of prostate cancer and was on a long-term catheter. However, the first ultrasound did not show any blockage, so we had to review the patient's history and examination to find other possible reasons for the AKI, such as kidney or pre-kidney issues, even though we still strongly suspected obstructive uropathy.

## Case presentation

A 79-year-old gentleman was admitted to our hospital with a blocked long-term urinary catheter. He had his urinary catheter changed twice in the ED over the past few days and has been passing clear urine since then. Notably, a week before this incident, he experienced several episodes of diarrhea during a trip to Norfolk, where he had eaten street food. He described diarrhea as "watery," with no mucus or blood in the stool. Upon admission to the hospital, he exhibited a deranged renal profile indicating Stage 1 AKI.

He is a known hypertensive who is being treated with indapamide and lisinopril, and he also has type 2 diabetes for which he takes alogliptin. He is on treatment for carcinoma of the prostate with 12 weekly leuprorelin injections and is on a long-term catheter. In addition to these medications, he also takes atorvastatin.

**Figure 1:**
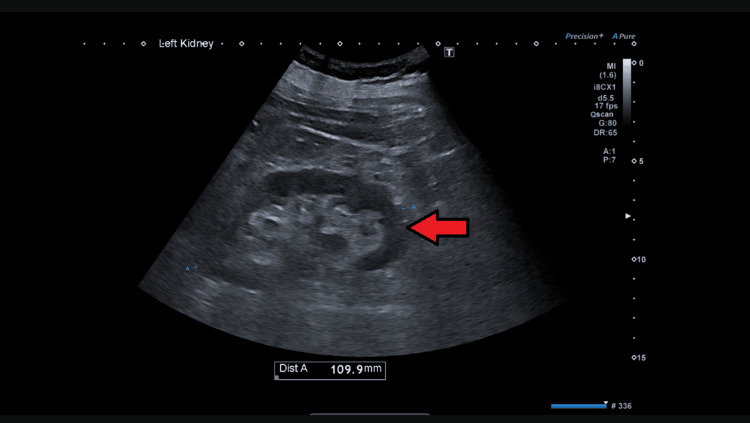
Ultrasound of the kidney, ureter, and bladder There was no significant hydronephrosis seen in the ultrasound. The left kidney measured around 10.9 cm. The red arrowhead indicates preserved corticomedullary differentiations.

**Figure 2:**
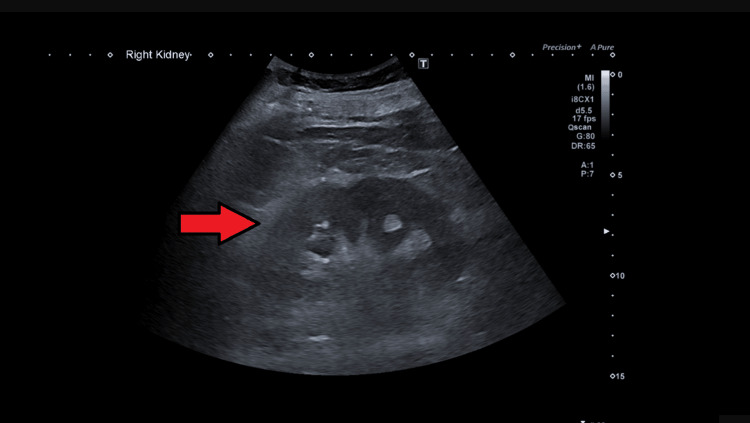
Ultrasound of the kidney, ureter, and bladder There was no significant hydronephrosis seen in the ultrasound. The right kidney measured around 10.6 cm. The red arrowhead indicates preserved corticomedullary differentiations.

Upon examination, he was fully alert and orientated. There was no raised jugular venous pressure, and he appeared euvolemic. His chest was clear, and no heart murmur was appreciated. There was no pedal edema seen. His abdomen was soft, and there was no palpable bladder or renal angle tenderness. His observational chart indicated that his blood pressure ranged from 90 to 100 mmHg. We initially used a differential diagnosis to rule out an obstructive cause of the AKI. We performed several bladder scans on admission, but they did not reveal urinary retention. We also requested a formal ultrasound scan of the abdomen (Figures 1, 2). The ultrasound revealed no evidence of obstruction or stones. Given the history of watery diarrhea, hypotension on examination, high inflammatory markers, and the absence of solid evidence of obstructive uropathy, our next strategy was to believe that a prerenal cause of AKI was the most likely culprit; therefore, we continued rehydration therapy, and we closely monitored his fluids and input and output charts.

Unfortunately, despite aggressive hydration, the renal profile and metabolic acidosis continued to deteriorate alarmingly (Table 1). During the admission, he had his urine catheter changed twice and continued to maintain a satisfactory urine output. We repeated numerous bladder scans; however, they did not show any evidence of urinary retention. On the third day of admission, he developed hypotension that was refractory to fluid resuscitation. Upon reviewing his blood investigations, we noticed that he had newly developed anemia and thrombocytopenia, as well as a mild elevation of bilirubin. Given that he had watery diarrhea before being admitted, our findings made us consider less common conditions such as hemolytic uremic syndrome (HUS) or thrombotic thrombocytopenic purpura (TTP). Upon examining the patient, he was fully alert and oriented; there were no obvious signs of neurological deficit or rash.

**Table 1:** Blood investigations before Tiemann tip insertion *Significant deranged results. Patient's history of watery diarrhea, hypotension, and unremarkable ultrasound kidney, ureter, and bladder scans suggest that a pre-renal cause of acute kidney injury (AKI) is the likely cause. However, the patient's renal profile continues to deteriorate despite fluid hydration, suggesting other differential diagnoses. A significant deterioration of renal profile, anemia, and raised bilirubin prompted us to think of a rarer cause of AKI such as hemolytic uremic syndrome (values highlighted in bold). The onset of anemia, thrombocytopenia, and hyponatremia suggested that the patient had urosepsis and dilutional hyponatremia (obstructive uropathy). CRP, C-reactive protein

Laboratory test	Date		
	08/12/2024 (Day 1: day of admission)	10/12/2024 (Day 3)	12/12/2024 (Day 5)
Sodium (133-146 mmol/L)	120	130*	130
Potassium (3.5-5.3 mmol/L)	4	3.8	3.7
Urea (2.5-7.8 mmol/L)	23	30.3*	32.4*
Creatinine (59-107 umol/L)	375	558*	618*
Hemoglobin (130-180 g/L)	132	108*	
White blood cells (4-11x10^9^/L)	15.3	9.6	
Platelets (150-400x10^9^/L)	90	91	
Total bilirubin (0-21 umol/L)	21	38*	
CRP (<5 mg/L)	211	155	

**Figure 3:**
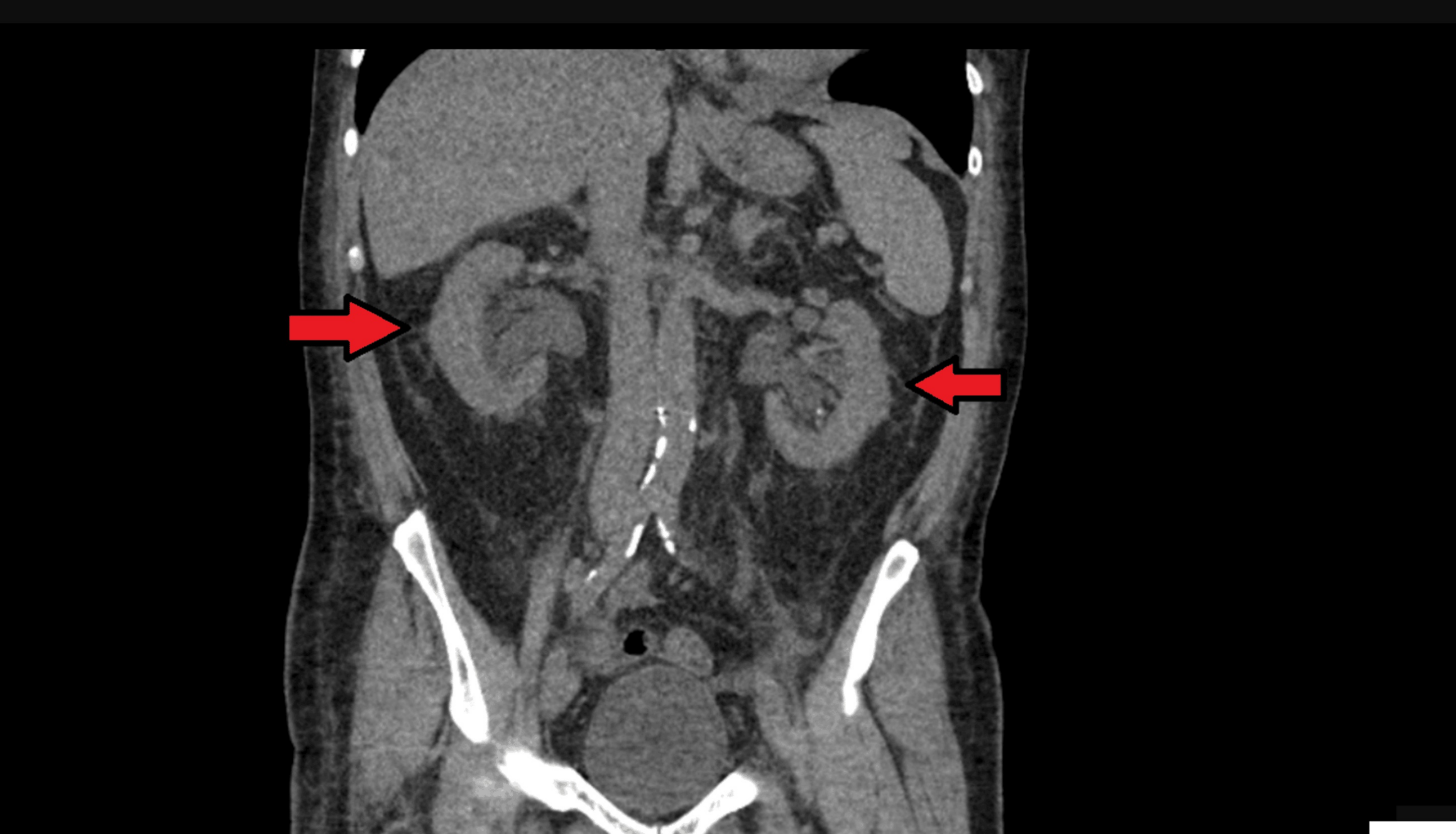
CT renal Bilateral arrowheads show evidence of severe hydronephrosis with moderate hydroureter bilaterally, without obvious cause. The kidneys are normal in size with severe hydronephrosis.

Keeping the rare but possible differentials in mind, we subsequently requested an urgent full blood picture, a hemolytic profile, and an autoimmune screen panel. A full blood picture did not show any evidence of hemolysis; other hemolysis workups came back as unremarkable. Despite fluid hydration, the patient continued to be persistently hypotensive. At this point, we decided to revisit our initial diagnosis for this patient, which was obstructive uropathy. It was the most plausible cause given his background of long-term catheter use and prostate carcinoma. Therefore, we requested an urgent CT scan of the kidney, ureter, and bladder in view of the diagnostic dilemma. The CT scan revealed severe hydronephrosis with bilateral moderate hydroureter (Figure 3), a likely obstructive cause of AKI initially missed in the ultrasound scan. We liaised urgently with our urology colleagues, who immediately reviewed the patient. They advised that a Tiemann tip silicone catheter be inserted, as it is very likely that a conventional urinary catheter was draining his bladder completely.

After a Tiemann tip urinary catheter was inserted, he drained as much as 6 L of urine (Table 2). Following the procedure, his renal profile and blood parameters significantly improved along with his clinical observations. The renal autoimmune profiles turned out to be unremarkable. The stool sample did not show *Escherichia coli* or Shiga toxin. After three days, his renal profile reverted back to baseline, his vital signs became stable, and he was discharged on medical advice afterwards (Table 3). 

**Table 2:** Fluid chart of the patient *Patient's significant change of fluid status. After a Tiemann Tip was exchanged with his usual urinary catheter, he had drained around 6 L of urine immediately. His renal profile subsequently returned to baseline in two days and he was discharged on medical advice.

Fluid chart	Date		
	09/12/2024	10/12/2024	11/12/2024 (After Tiemann tip insertion)
Input (mL)	1850	1575	1800
Output (mL)	2000	476	*6170
Total (mL)	-150	1039	*-4370

**Table 3:** Laboratory test (stool, hemolytic, and renal screen) after Tiemann tip insertion *Improving renal profile after a Tiemann tip insertion. Other blood investigations show unremarkable hemolytic screen, stool sample results, and renal screen. His renal profile was back to baseline; he was discharged on medical advice. ANA, antinuclear antibody; CRP, C-reactive protein; FBP, full blood picture; GBM, glomerular basement membrane; LDH, lactate dehydrogenase; PLA2R, phospholipase A2 receptor; WBC, white blood cell

Laboratory test	Date	
	12/12/2024	13/12/2024 (Date of discharge)
Sodium (133-146 mmol/L)	141	141
Potassium (3.5-5.3 mmol/L)	3.7	4
Urea (2.5-7.8 mmol/L)	*11.9	*5.8
Creatinine (59-107 umol/L)	*122	*85
Hemoglobin (130-180 g/L)	124	119
White blood cells (4-11x10^9^/L)	7.1	8.3
Platelets (150-400x10^9^/L)	197	208
Total bilirubin (0-21 umol/L)	18	20
CRP (<5 mg/L)		30
*Clostridium difficile* antigen and toxin	Negative	
*Salmonella* stool culture	Negative	
*Shigella* stool culture	Negative	
*Campylobacter* stool culture	Negative	
*E. coli* O157 stool culture	Negative	
Blood film (FBP)	No evidence of hemolysis	
Direct antiglobulin	Positive	
IgA (0.8-4 g/L)	1.7	
IgG (6-16 g/L)	8.3	
IgM (0.5-2 g/L)	1.6	
Complement C3 (0.9-1.8 g/L)	1.1	
Complement C4 (0.14-0.54 g/L)	<0.1	
Anti-PLA2R antibody (0-14 kU/L)	<2	
Anti-GBM antibody (7-10 U/mL)	<0.8	
LDH (240-400 U/L)	369	
Reticulocyte count		
Antinuclear antibody (0.1-0.69)	0.4	

## Discussion

Although AKI is a common condition encountered in daily clinical practice, sometimes determining its underlying cause can be challenging, as illustrated by this case presentation. We discussed a 75-year-old male on a long-term urinary catheter who presented to us with AKI [2]. Initially, ruling out an obstructive cause was our main differential diagnosis. However, the initial negative bladder scans, negative formal ultrasound kidney, ureter, and bladder results, and persistent hypotension despite fluid resuscitation led us to consider both pre-renal and renal causes for the AKI [4-6]. Most clinical cases are not as straightforward as in our medical literature, and therefore, we found it quite challenging to determine the exact nature of the AKI.

His worsening renal profile, despite fluid challenges, antibiotics, and supportive management, pushed us to rethink. Is there anything we may have missed? While ultrasound is quite sensitive in identifying hydronephrosis, in certain clinical scenarios, such as early obstructive uropathy, operator dependence, or suboptimal visualization, its sensitivity may be reduced [7]. Therefore, in cases of diagnostic uncertainty and clinical deterioration, with a high degree of suspicion for an obstructive uropathy, a CT scan should be promptly considered to rule out obstruction [8]. This case serves as a reminder that even seemingly routine medical conditions can evolve unpredictably if appropriate treatment is not offered timely.

On the other hand, we should always remain vigilant in thinking of a broader differential diagnosis, even if it is a rare and life-threatening cause. TTP and HUS are rare diseases that both work in a similar way, called thrombotic microangiopathy (TMA). TMA occurs when small blood vessels get blocked with microthrombi, leading to low platelet counts, destruction of red blood cells, and failure of multiple organs [9]. Even though the patient did not show clear signs of organ failure besides AKI or neurological issues, the presence of anemia, low platelet counts, and high bilirubin levels, along with a quick decline in kidney function, made us want to look deeper, since the treatment could be very different and more specific than usual care. For example, we could use plasma exchange or administer biological agents such as eculizumab. The ongoing urosepsis in this case likely contributed to the development of anemia and thrombocytopenia, as well as the elevated bilirubin [10].

With the establishment of an obstructive uropathy, we were able to resort to the most appropriate treatment, which was addressing the bladder outflow obstruction by changing the catheter. However, in this case, even this proved challenging, as the traditional urinary catheter was unable to completely relieve the obstruction. Therefore, with the advice from urology specialists, a Tiemann tip catheter was inserted, relieving the urinary retention. Following this, the blood parameters improved significantly, normalizing within a few days. 

## Conclusions

This case highlights the importance of maintaining a high index of clinical suspicion in our daily medical practice. While initial history and examination pointed toward an obstructive cause for the AKI, the lack of evidence from the initial sonography made us reconsider other causes of AKI, such as pre-renal and renal causes. The rapid clinical and biochemical deterioration prompted us to broaden our differential diagnoses. Ultimately, further imaging studies and excellent clinical judgement led us to a diagnosis of obstructive uropathy, despite earlier negative imaging.

Additionally, this case teaches us to remain vigilant regarding rare but serious conditions in our practice. A thorough and detailed history, focused clinical examination, and appropriate investigations, along with a high index of clinical suspicion, were the keys to achieving a positive outcome in this complex clinical case. 

## References

[1] Fliser D, Laville M, Covic A, Fouque D, Vanholder R, Juillard L, Van Biesen W (2012). A European Renal Best Practice (ERBP) position statement on the Kidney Disease Improving Global Outcomes (KDIGO) clinical practice guidelines on acute kidney injury: part 1: definitions, conservative management and contrast-induced nephropathy. Nephrol Dial Transplant.

[2] Khwaja A (2012). KDIGO clinical practice guidelines for acute kidney injury. Nephron Clin Pract.

[3] Lameire N, Biesen WV, Vanholder R (2005). Acute renal failure. Lancet.

[4] Gottlieb RH, Weinberg EP, Rubens DJ (1997). Renal sonography: can it be used more selectively in the setting of an elevated serum creatinine level?. Am J Kidney Dis.

[5] Sibley S, Roth N, Scott C, Rang L, White H, Sivilotti ML, Bruder E (2020). Point-of-care ultrasound for the detection of hydronephrosis in emergency department patients with suspected renal colic. Ultrasound J.

[6] Al-Balushi A, Al-Shibli A, Al-Reesi A (2022). The accuracy of point-of-care ultrasound performed by emergency physicians in detecting hydronephrosis in patients with renal colic. Sultan Qaboos Univ Med J.

[7] Ather MH, Jafri AH, Sulaiman MN (2004). Diagnostic accuracy of ultrasonography compared to unenhanced CT for stone and obstruction in patients with renal failure. BMC Med Imaging.

[8] Nester CM, Feldman DL, Burwick R (2024). An expert discussion on the atypical hemolytic uremic syndrome nomenclature-identifying a road map to precision: a report of a National Kidney Foundation Working Group. Kidney Int.

[9] Page EE, Kremer Hovinga JA, Terrell DR, Vesely SK, George JN (2017). Thrombotic thrombocytopenic purpura: diagnostic criteria, clinical features, and long-term outcomes from 1995 through 2015. Blood Adv.

[10] (2025). UpToDate: Sepsis syndromes in adults: epidemiology, definitions, clinical presentation, diagnosis, and prognosis. https://www.uptodate.com/contents/sepsis-syndromes-in-adults-epidemiology-definitions-clinical-presentation-diagnosis-and-prognosis?search=bicytopenia%20sepsis&source=search_result&selectedTitle=8~150&usage_type=default&display_rank=7.

